# Bone marrow examination in newly diagnosed Hodgkin's disease: current practice in the United Kingdom.

**DOI:** 10.1038/bjc.1995.43

**Published:** 1995-01

**Authors:** M. R. Howard, P. R. Taylor, H. H. Lucraft, M. J. Taylor, S. J. Proctor

**Affiliations:** Department of Haematology, Royal Victoria Infirmary, Newcastle upon Tyne, UK.

## Abstract

In the UK Hodgkin's disease is usually treated by either clinical oncologists or haematologists. A national study of the performance of bone marrow examination in newly diagnosed Hodgkin's disease was undertaken to establish current practice. A total of 620 questionnaires were despatched, and replies were received from 60% of consultants (45% of clinical oncologists and 70% of haematologists). Bone marrow examination was performed in all new cases significantly more often by haematologists than by clinical oncologists (74% vs 40%, P < 0.001). Among haematologists, there was no correlation between the number of new patients seen annually and practice, however clinical oncologists were even less likely to perform routine bone marrow biopsies if they saw more than ten patients per year (P < 0.02). Where bone marrow examination was performed selectively, the most common criteria used were peripheral blood cytopenia and advanced-stage disease. These criteria were applied in the same way by both clinical oncologists and haematologists. Bone marrow biopsy, an invasive and often painful procedure, is currently performed more frequently in Hodgkin's disease than can be recommended on the basis of recent studies in the literature and associated guidelines. There is a significant difference in practice between clinical oncologists and haematologists, and this raises the wider issue of the influence of hospital specialisation on patient management.


### Bone marrow examination in newly diagnosed Hodgkin's disease: current practice in the United Kingdom

MR Howard[1], PRA Taylor[1], HH Lucraft[2], MJ Taylor[3] and SJ Proctor[1]

*Departments of [1]Haematology and [2]Clinical Oncology, Royal Victoria Infirmary, Queen Victoria Road, Newcastle upon Tyne NE1 4LP, UK; [3]Gonville and Caius College, Cambridge CB2 1TA, UK.*

**Summary** In the UK Hodgkin's disease is usually treated by either clinical oncologists or haematologists. A national study of the performance of bone marrow examination in newly diagnosed Hodgkin's disease was undertaken to establish current practice. A total of 620 questionnaires were despatched, and replies were received from 60% of consultants (45% of clinical oncologists and 70% of haematologists). Bone marrow examination was performed in all new cases significantly more often by haematologists than by clinical oncologists (74% *vs* 40%, $P<0.001$). Among haematologists, there was no correlation between the number of new patients seen annually and practice, however clinical oncologists were even less likely to perform routine bone marrow biopsies if they saw more than ten patients per year ($P<0.02$). Where bone marrow examination was performed selectively, the most common criteria used were peripheral blood cytopenia and advanced-stage disease. These criteria were applied in the same way by both clinical oncologists and haematologists. Bone marrow biopsy, an invasive and often painful procedure, is currently performed more frequently in Hodgkin's disease than can be recommended on the basis of recent studies in the literature and associated guidelines. There is a significant difference in practice between clinical oncologists and haematologists, and this raises the wider issue of the influence of hospital specialisation on patient management.

**Keywords:** Hodgkin's disease; bone marrow

Hodgkin's disease is a well-characterised lymphoma with a widely accepted histological classification and staging system (Urba *et al.*, 1992). In the UK patients with the disease are normally referred for assessment and treatment to either clinical oncologists or haematologists. Over the last 20 years many aspects of management have changed. The introduction of new imaging technology has led to a revision of priorities for routine investigation with a reduced requirement for invasive procedures such as laparotomy. It has been the authors' impression that there is currently little consensus as to the importance of bone marrow examination in newly diagnosed patients.

This study of the practice of examining the bone marrow in newly diagnosed Hodgkin's disease was undertaken to establish current practice in the UK. We aimed to determine the degree of variability in the frequency with which this investigation is performed, the extent of conformity with published guidelines and whether there was significant difference in practice between clinical oncologists and haematologists.

### Methods

Consultant clinical oncologists and haematologists currently practising in the UK were identified from records of the Royal College of Pathology and the Faculty of Clinical Oncology of the Royal College of Radiologists. Each consultant was sent the questionnaire illustrated in the appendix. Those not replying were prompted with a further questionnaire 4 weeks later.

### *Statistical methods*

Comparisons between groups were made using the chi-square test or Fisher's exact test.

Correspondence: MR Howard, Department of Haematology, York District Hospital, Wiggington Road, York YO3 7HE, UK

Received 3 June 1994; revised 24 August 1994; accepted 24 August 1994

### Results

A total of 620 questionnaires were despatched, 260 to clinical oncologists and 360 to haematologists. Following prompting, 370 (60%) replies were received, 117 (45%) from clinical oncologists and 253 (70%) from haematologists. Of those responding to the questionnaire, 17 (15%) clinical oncologists and 67 (26%) haematologists did not see patients with Hodgkin's disease and were excluded from further analysis.

Thirty-one (11%) consultants saw more than ten cases per year, 129 (45%) 5-10 cases and 125 (44%) fewer than five cases. Haematologists were more likely than clinical oncologists to see fewer than five new referrals per annum ($P<0.001$).

Routine bone marrow examination in all patients was performed significantly more often by haematologists than by clinical oncologists (74% *vs* 40%, $P<0.001$). Among haematologists the decision to perform bone marrow examination in patients selectively was not affected by the number of patients with Hodgkin's disease seen. However, clinical oncologists were less likely to perform a routine bone marrow if they saw more than ten patients per year than if they saw fewer than ten patients ($0.02>P>0.01$).

Where bone marrow examination was performed selectively the criteria used are illustrated in Table I. The most common criteria used to decide upon bone marrow examination were peripheral blood cytopenia (particularly thrombocytopenia), advanced stage disease and logistical considerations such as the likelihood of future transplantation or entry into a study protocol. These criteria were applied in the same way by both clinical oncologists and haematologists.

The number of clinicians performing bone marrow examination in all patients did not differ in different regions. Most clinicians performed bone marrow trephines at one site, only 9% at two sites and less than 1% at three sites with no difference between specialties. Bone marrow trephines were reviewed by haematologists alone in 40% of cases. A combined review with a histopathologist was performed in 50% and histopathologists alone performed review in 10%. No clinicians had changed their reasons for bone marrow examination over the previous 2 years.

Table I Criteria used where bone marrow examination performed selectively

| Criteria for bone marrow examination | Clinical oncologists | Haematologists | All |
|---|---|---|---|
| Thrombocytopenia | 53 (93%) | 46 (94%) | 99 (94%) |
| Leucopenia | 51 (89%) | 43 (88%) | 94 (89%) |
| Hb outside normal range | 41 (72%) | 37 (76%) | 78 (74%) |
| Other peripheral blood abnormality | 31 (54%) | 27 (55%) | 58 (55%) |
| High erythrocyte sedimentation rate/plasma viscosity | 15 (26%) | 7 (14%) | 22 (21%) |
| Advanced-stage disease | 37 (65%) | 26 (53%) | 63 (59%) |
| B symptoms | 32 (56%) | 17 (35%) | 49 (46%) |
| Requirement of study protocol | 44 (77%) | 32 (65%) | 76 (72%) |
| Request by other consultant | 7 (12%) | 18 (37%) | 25 (24%) |
| Patient eligible for future auto/allograft | 31 (54%) | 29 (59%) | 60 (57%) |
| Other | 4 (7%) | 5 (10%) | 9 (8%) |

### Discussion

It is well recognised that marrow infiltration by malignant cells occurs in approximately 10% of cases of newly presenting Hodgkin's disease (Bartl *et al.*, 1982; Schmid *et al.*, 1992; Stark *et al.*, 1992; Urba *et al.*, 1992). This is closely associated with advanced clinical stage, and is found in only 1–2% of patients otherwise staged as I or II (Bartl *et al.*, 1982). In biopsies not infiltrated by malignancy other abnormalities, most commonly a mixed inflammatory cell infiltrate may be seen, but these non-specific changes appear to have limited prognostic significance and do not influence the staging or treatment of the disease (Bartl *et al.*, 1982). A study of 613 cases of Hodgkin's disease in the UK (Macintyre *et al.*, 1987) found that the bone marrow biopsy result affected the mode of treatment in less than 1% of patients. A French study indicated it was only contributory in patients with 'B' symptoms (Eghbali *et al.*, 1993). In view of the rarity of marrow infiltration in early-stage disease and the limited impact on patient management, recent guidelines have generally recommended reserving bone marrow biopsy for selected patients. Thus the Cotswold Meeting Committee suggested bone marrow examination be restricted to patients with stage III–IV or adverse stage II disease (Lister *et al.*, 1989) and the British National Lymphoma Investigation protocols designate the procedure as 'non-mandatory' unless autotransplantation is planned (Macintyre *et al.*, 1987).

Our study demonstrates that, despite these guidelines, the majority of patients with newly presenting Hodgkin's disease in the UK have a bone marrow biopsy performed irrespective of stage or other criteria. Haematologists are significantly more likely than clinical oncologists to biopsy marrow routinely. Whereas haematologists' practice appears not to be influenced by the number of cases of Hodgkin's disease they see annually, clinical oncologists with greater experience of treating Hodgkin's disease were less likely to biopsy routinely. Such widespread marrow examination in all cases suggests that many patients are having an invasive investigation with only a minimal chance of the result influencing their management.

Where bone marrow examination was performed selectively, both groups of clinicians had the same priorities. There was a uniform lack of concordance with the Cotswold guidelines (Lister *et al.*, 1989), more emphasis being placed on peripheral blood abnormalities than the clinical stage of disease. The majority of clinicians only sampled at one site, despite evidence that where bone marrow examination is indicated bilateral biopsies significantly increase the probability of detecting infiltration (Bartl *et al.*, 1982).

The difference in practice between clinical oncologists and haematologists is of particular interest. With the increasing fragmentation of hospital medicine clinicians within different specialities have different clinical experience and postgraduate training. Ease of access to investigational and treatment facilities is also variable. Patients with identical clinical characteristics may be referred to different specialties. The demonstrable difference in approach to bone marrow examination in Hodgkin's disease between clinical oncologists and haematologists is likely to have parallels in other diseases and other specialties. Such variability in the management of a discrete clinical problem by different specialties is undesirable, as rational medical management should presumably be based on clinical characteristics rather than practitioner-associated factors.

### References

BARTL R, FRISCH B, BUCKHARDT R, HUHN D AND PAPPENBERGER R. (1982). Assessment of bone marrow histology in Hodgkin's disease: correlation with clinical factors. *Br. J. Haematol.*, **51**, 345–360.

EGHBALI H, BONICHON F, BONNEL C, SOUBEYRON P, DE MASCAREL I AND HOERNI B. (1993). Clinical features predicting bone marrow involvement in Hodgkin's disease: attempt to avoid unnecessary investigations in favourable presentation. *5th International Conference on Malignant Lymphomas.*

LISTER TA, CROWTHER D, SUTCLIFFE SB, GLATSTEIN E, CANELLOS GP, YOUNG RC, ROSENBERG SA, COLTMAN CA AND TUBIANA M. (1989). Report of a committee convened to discuss the evaluation and staging of patients with Hodgkin's disease: Cotswolds Meeting. *J. Clin. Oncol.*, **7**(11), 1630–1636.

MACINTYRE EA, VAUGHAN HUDSON B, LINCH DC, VAUGHAN HUDSON G AND JELLIFFE A.M. (1987). The value of bone marrow trephine biopsy in Hodgkin's disease. *Eur. J. Haematol.*, **39**, 66–70.

SCHMID C AND ISAACSON PG. (1992). Bone marrow trephine biopsy in lymphoproliferative disease. *J. Clin. Pathol.*, **45**, 745–750.

STARK AN AND ISAACSON PG. (1992). Staging and follow up of patients with lymphoproliferative disorder. *J. Clin. Pathol.*, **46**, 286.

URBA WJ AND LONGO DL. (1992). Hodgkin's disease. *N. Engl. J. Med.*, **326**, 678–687.

**Appendix I**

1. Do you have responsibility for the care/management of patients with Hodgkin's disease?

☐ Yes ☐ No

If **no**, there is no need to answer any further questions. Please could you return this form to complete our records.

If **yes**, please complete the remaining questions.

2. How many *new* patients with Hodgkin's disease do you see in a year?

☐ <5 ☐ 5–10 ☐ 10+

3. Do you examine the bone marrow in all your patients with Hodgkin's disease at presentation?

☐ Yes ☐ No

4. If no, which of the following would make you consider performing a bone marrow at diagnosis?

☐ Hb outside normal range
☐ Leucopenia
☐ Thrombocytopenia
☐ Other peripheral blood abnormality
☐ High ESR/plasma viscosity
☐ Advanced stage disease (specify):
☐ B symptoms
☐ Requirement of study protocol
☐ Request by other consultant
☐ Patient eligible for future auto/allograft
☐ Other (specify):

5. When you perform a bone marrow do you routinely biopsy?

☐ One site ☐ Two sites ☐ Four sites

6. Who reviews your trephine biopsies?

☐ Haematologist ☐ Histologist

☐ Both of above ☐ Other (specify):

7. Have you changed your policy for doing bone marrow examinations in the last two years (since 01.01.91). If so, please could you tell us why?


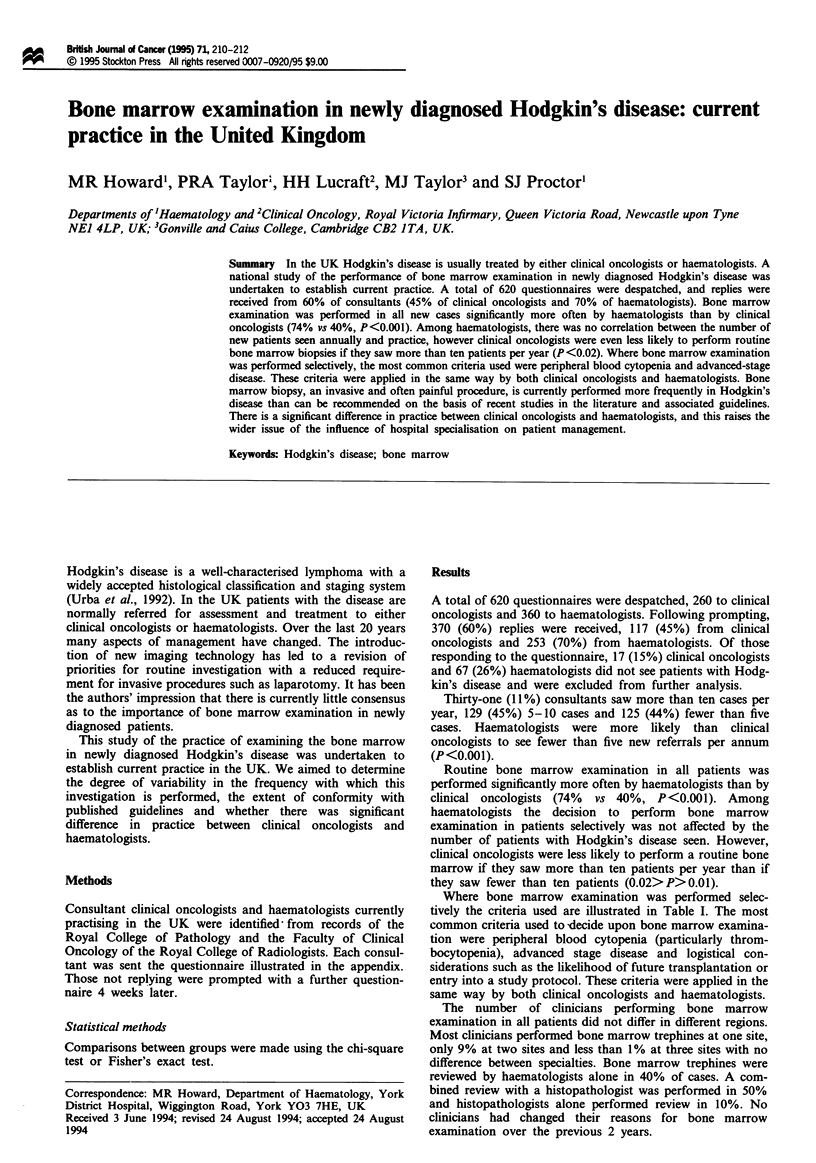

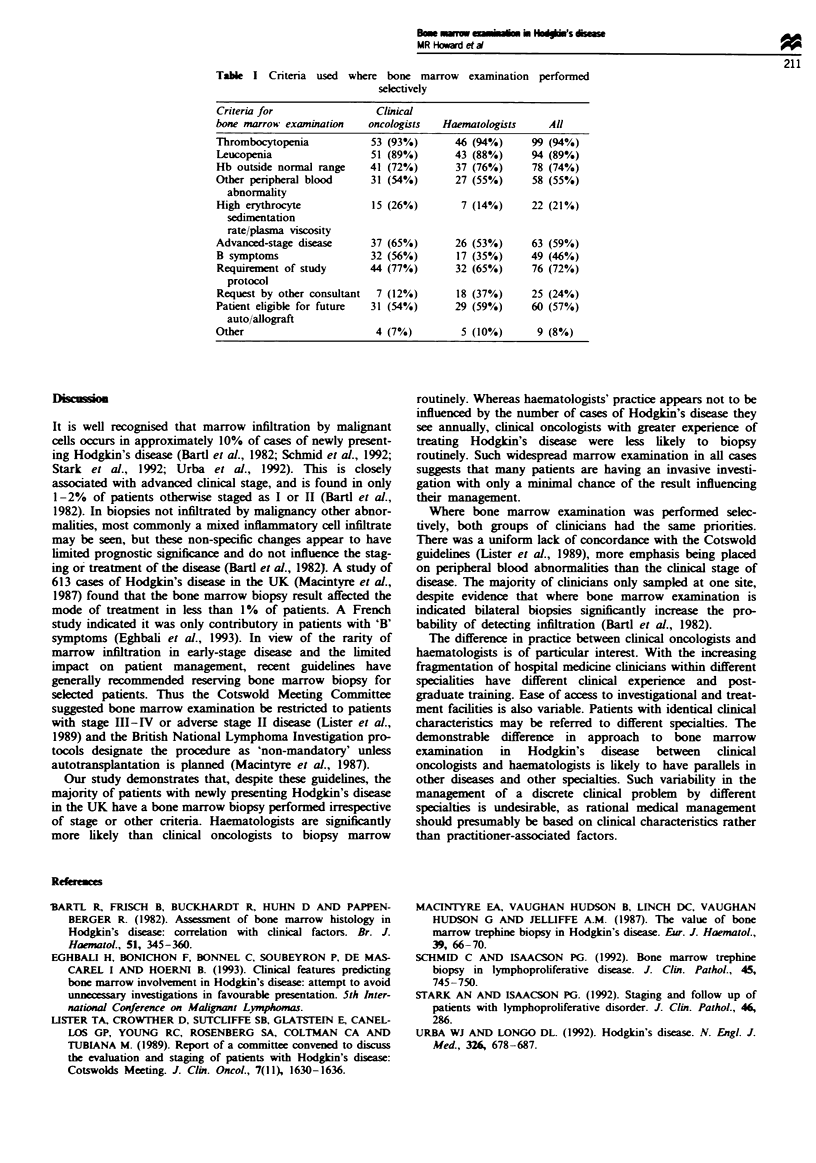

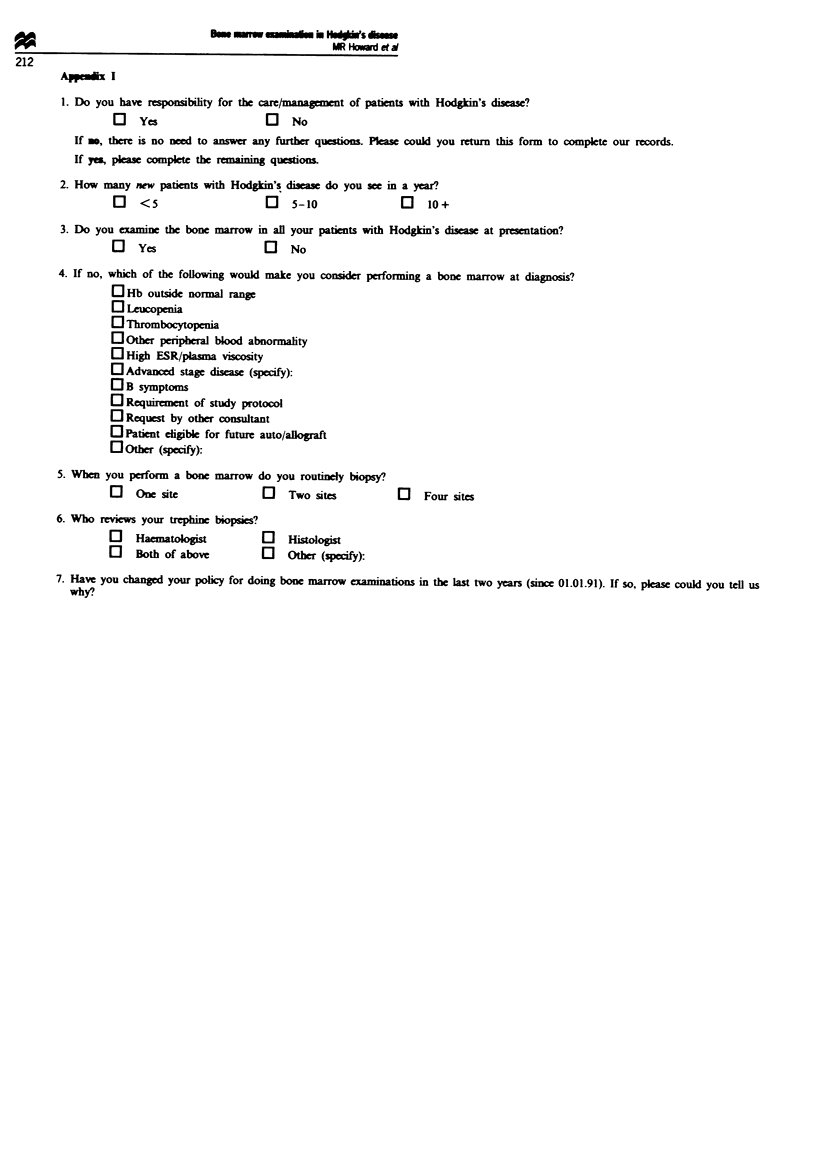


## References

[OCR_00237] Bartl R., Frisch B., Burkhardt R., Huhn D., Pappenberger R. (1982). Assessment of bone marrow histology in Hodgkin's disease: correlation with clinical factors.. Br J Haematol.

[OCR_00400] Delic J. I., Bush C., Peckham M. J. (1986). Protection from procarbazine-induced damage of spermatogenesis in the rat by androgen.. Cancer Res.

[OCR_00403] Drasga R. E., Einhorn L. H., Williams S. D., Patel D. N., Stevens E. E. (1983). Fertility after chemotherapy for testicular cancer.. J Clin Oncol.

[OCR_00408] Fairley K. F., Barrie J. U., Johnson W. (1972). Sterility and testicular atrophy related to cyclophosphamide therapy.. Lancet.

[OCR_00413] Flickinger C. J. (1977). Effects of clompihene on the structure of the testis, epididymis and sex accessory glands of the rat.. Am J Anat.

[OCR_00417] Glode L. M., Robinson J., Gould S. F. (1981). Protection from cyclophosphamide-induced testicular damage with an analogue of gonadotropin-releasing hormone.. Lancet.

[OCR_00422] Heller C. G., Rowley M. J., Heller G. V. (1969). Clomiphene citrate: a correlation of its effect on sperm concentration and morphology, total gonadotropins, ICSH, estrogen and testosterone excretion, and testicular cytology in normal men.. J Clin Endocrinol Metab.

[OCR_00429] Hollinger M. A. (1970). Effect of clomiphene on testicular protein synthesis in vitro.. Biochem Pharmacol.

[OCR_00440] Hollinger M. A., Hwang F. (1972). Effect of in vivo and in vitro administration of clomiphene on RNA synthesis in rat testis.. Arch Int Pharmacodyn Ther.

[OCR_00452] Johnson D. H., Linde R., Hainsworth J. D., Vale W., Rivier J., Stein R., Flexner J., Van Welch R., Greco F. A. (1985). Effect of a luteinizing hormone releasing hormone agonist given during combination chemotherapy on posttherapy fertility in male patients with lymphoma: preliminary observations.. Blood.

[OCR_00445] Jégou B., Velez de la Calle J. F., Bauché F. (1991). Protective effect of medroxyprogesterone acetate plus testosterone against radiation-induced damage to the reproductive function of male rats and their offspring.. Proc Natl Acad Sci U S A.

[OCR_00458] Karashima T., Zalatnai A., Schally A. V. (1988). Protective effects of analogs of luteinizing hormone-releasing hormone against chemotherapy-induced testicular damage in rats.. Proc Natl Acad Sci U S A.

[OCR_00469] Lewis R. W., Dowling K. J., Schally A. V. (1985). D-Tryptophan-6 analog of luteinizing hormone-releasing hormone as a protective agent against testicular damage caused by cyclophosphamide in baboons.. Proc Natl Acad Sci U S A.

[OCR_00250] Lister T. A., Crowther D., Sutcliffe S. B., Glatstein E., Canellos G. P., Young R. C., Rosenberg S. A., Coltman C. A., Tubiana M. (1989). Report of a committee convened to discuss the evaluation and staging of patients with Hodgkin's disease: Cotswolds meeting.. J Clin Oncol.

[OCR_00255] Macintyre E. A., Vaughan Hudson B., Linch D. C., Vaughan Hudson G., Jelliffe A. M. (1987). The value of staging bone marrow trephine biopsy in Hodgkin's disease.. Eur J Haematol.

[OCR_00479] Meistrich M. L., Wilson G., Ye W. S., Kurdoglu B., Parchuri N., Terry N. H. (1994). Hormonal protection from procarbazine-induced testicular damage is selective for survival and recovery of stem spermatogonia.. Cancer Res.

[OCR_00495] Morris I. D., Bardin C. W., Gunsalus G., Ward J. A. (1990). Prolonged suppression of spermatogenesis by oestrogen does not preserve the seminiferous epithelium in procarbazine-treated rats.. Int J Androl.

[OCR_00485] Morris I. D., Shalet S. M. (1990). Protection of gonadal function from cytotoxic chemotherapy and irradiation.. Baillieres Clin Endocrinol Metab.

[OCR_00501] Nseyo U. O., Huben R. P., Klioze S. S., Pontes J. E. (1985). Protection of germinal epithelium with luteinizing hormone-releasing hormone analogue.. J Urol.

[OCR_00506] Papadopoulos I. (1991). LHRH analogues do not protect the germinal epithelium during chemotherapy. An experimental animal investigation.. Urol Res.

[OCR_00511] Parchuri N., Wilson G., Meistrich M. L. (1993). Protection by gonadal steroid hormones against procarbazine-induced damage to spermatogenic function in LBNF1 hybrid rats.. J Androl.

[OCR_00519] Pennisi A. J., Grushkin C. M., Lieberman E. (1975). Gonadal function in children with nephrosis treated with cyclophosphamide.. Am J Dis Child.

[OCR_00524] Roeser H. P., Stocks A. E., Smith A. J. (1978). Testicular damage due to cytotoxic drugs and recovery after cessation of therapy.. Aust N Z J Med.

[OCR_00529] Schilsky R. L., Lewis B. J., Sherins R. J., Young R. C. (1980). Gonadal dysfunction in patients receiving chemotherapy for cancer.. Ann Intern Med.

[OCR_00261] Schmid C., Isaacson P. G. (1992). Bone marrow trephine biopsy in lymphoproliferative disease.. J Clin Pathol.

[OCR_00266] Stark A. N. (1993). Staging and follow up of patients with lymphoproliferative disorder.. J Clin Pathol.

[OCR_00271] Urba W. J., Longo D. L. (1992). Hodgkin's disease.. N Engl J Med.

[OCR_00532] Velez de la Calle J. F., Jégou B. (1990). Protection by steroid contraceptives against procarbazine-induced sterility and genotoxicity in male rats.. Cancer Res.

[OCR_00537] Ward J. A., Robinson J., Furr B. J., Shalet S. M., Morris I. D. (1990). Protection of spermatogenesis in rats from the cytotoxic procarbazine by the depot formulation of Zoladex, a gonadotropin-releasing hormone agonist.. Cancer Res.

[OCR_00544] Waxman J. (1987). Preserving fertility in Hodgkin's disease.. Baillieres Clin Haematol.

[OCR_00548] Weissenberg R., Dar Y., Lunenfeld B. (1992). The effect of clomiphene citrate and its Zu or En isomers on the reproductive system of the immature male rat.. Andrologia.

[OCR_00553] Whitehead E., Shalet S. M., Blackledge G., Todd I., Crowther D., Beardwell C. G. (1982). The effects of Hodgkin's disease and combination chemotherapy on gonadal function in the adult male.. Cancer.

[OCR_00392] da Cunha M. F., Meistrich M. L., Nader S. (1987). Absence of testicular protection by a gonadotropin-releasing hormone analogue against cyclophosphamide-induced testicular cytotoxicity in the mouse.. Cancer Res.

